# Stigma in Tuberculosis: Time to Act on an Important and Largely Unaddressed Issue

**DOI:** 10.7759/cureus.61964

**Published:** 2024-06-08

**Authors:** Sankalp Yadav

**Affiliations:** 1 Medicine, Shri Madan Lal Khurana Chest Clinic, New Delhi, IND

**Keywords:** treatment delays, tuberculosis related stigma, physical and psychological well-being, stigma, tuberculosis

## Abstract

Tuberculosis has afflicted mankind for centuries, serving as a prevalent cause of morbidity and mortality in high-burden countries. Despite ongoing efforts in tuberculosis control, critical issues, such as the psychological impact of the disease, often go unaddressed. This editorial sheds light on a crucial aspect of tuberculosis: the stigma associated with the infection and its profound impact. Additionally, it offers suggestions to overcome the shortcomings in the psychological management of patients.

## Editorial

Tuberculosis has been known for ages and is a significant threat to healthcare globally. It comes in second after coronavirus disease 2019 in terms of morbidity and mortality, and it has all the potential to reclaim its top spot in the aftermath of the pandemic of severe acute respiratory syndrome coronavirus 2 [1]. It is rampant in the countries of Southeast Asia (45%), Africa (23%), and the Western Pacific (18%), with smaller shares in the Eastern Mediterranean (8.1%), the Americas (2.9%), and Europe (2.2%) and has affected millions in these high-burden settings [2]. The disease not only affects the physical well-being of the patient but also has profound effects on the mental and psychological health of the sufferers [3].

Patients with tuberculosis encounter an enduring and deeply ingrained stigma, just like those with leprosy, HIV/AIDS, and other immunological deficiencies. Goffman described stigma as “an attribute that is deeply discrediting” that demeans people “from a whole and usual person to a tainted, discounted one [4]." Tuberculosis patients may experience profound consequences from this in a variety of social contexts, including the home, workplace, and community. Studies conducted in various settings have revealed that between 42% and 82% of patients suffering from tuberculosis experience stigma. Thereby, stigma associated with the disease is one of the biggest obstacles to tuberculosis prevention and control today [3].

According to the World Health Organization, stigma is a mark of shame, humiliation, or disapproval that causes someone to be rejected, subjected to discrimination, and barred from engaging in many societal activities. Stigma seriously impairs the tuberculosis response and damages the lives and health of those who experience it. Fear of losing one's work, relationships, or house or school due to tuberculosis makes people less likely to get tested and treated, which exacerbates an already challenging prognosis [5].

Further, the conditions that are frequently linked to tuberculosis may aggravate stigma, for example, HIV, poverty, drug and alcohol abuse, homelessness, past criminal activity, and refugee or night shelter status. Some subpopulations experience the stigma of tuberculosis more intensely than others such as women, refugees, those living in rural regions, and those with lower levels of education. Individuals who face discrimination may experience social isolation, especially in smaller areas where entire families may be disapproved of. Those who contract tuberculosis may be declared unfit for marriage or divorce because women are frequently held responsible for the disease's spread.

Stigma could be further divided into three categories, as mentioned in Table 1 [6].

**Table 1:** Categories of stigma Source: [6]

Stigma	Impact
Experienced stigma	The experience of exclusion and/or discrimination
Anticipated stigma	The perception, expectation, and/or fear of stigma
Internalized stigma	A loss of self-esteem, dignity, fear, and/or shame

Overall, the effects of the stigma are unrelenting, as diagnostic delays could result in severe forms of disease. Moreover, developing drug resistance is also an impending threat in such circumstances. The situation becomes graver when these patients, due to fear of being recognized and to avoid discrimination, avoid taking precautions like wearing masks in public, ultimately spreading the disease. Oftentimes, they are in self-denial and, due to fear, do not visit health facilities. A majority of patients are daily wage workers or laborers working in factories in close proximity to many other workers, thereby exposing them to infection. Children often do not follow precautions, and due to fear of stigma and discrimination, they attend their classes without masks and proper coughing hygiene. Additionally, stigmatized patients often skip their medications, as this requires them to visit healthcare facilities. This could result in lost-to-follow-up cases due to non-compliance with treatment adherence, which could later present with drug-resistant forms of the disease [6].

In international tuberculosis prevention and control efforts, stigma has largely remained a low-priority issue [6]. Yet, in recent years, there has been remarkable awareness related to this aspect of tuberculosis care [3]. Efforts to keep a check on the stigma associated with the disease involve focusing on interventions aimed at reducing the stigma associated with tuberculosis: raising community awareness; counseling patients on problem-solving and emotional skills; developing culturally sensitive and scientifically sound media messages; offering financial support to patients; and improving the traits of healthcare professionals such as empathy, concern, respect for the patient, and cultural sensitivity [7]. In countries like India, government agencies, in collaboration with private partners, have laid down a training curriculum strategy to end the stigma and discrimination associated with tuberculosis [5].

To conclude, stigma associated with tuberculosis is a significant social determinant of health, as it not only impacts the psychological well-being of the patient but could also affect the socioeconomic stability of these patients. Fear of losing jobs, life partners, friends, education, etc. could result in deeper impacts on the individuals, which could last beyond the antituberculous treatment durations. Hence, it is imperative that all stakeholders consider addressing this issue with utmost importance and devotion. Having empathy with the patients who are already stigmatized could help them overcome the effects of tuberculosis.
